# Feasibility and Short-Term Toxicity of a Consecutively Delivered Five Fraction Stereotactic Body Radiation Therapy Regimen in Early-Stage Breast Cancer Patients Receiving Partial Breast Irradiation

**DOI:** 10.3389/fonc.2022.901312

**Published:** 2022-07-08

**Authors:** Yilan Liu, Christopher Veale, Diana Hablitz, Helen Krontiras, Allison Dalton, Korie Meyers, Michael Dobelbower, Rachael Lancaster, Markus Bredel, Catherine Parker, Kimberly Keene, Evan Thomas, Drexell Boggs

**Affiliations:** ^1^ Department of Radiation Oncology, University of Alabama at Birmingham, Birmingham, AL, United States; ^2^ Department of Surgery, University of Alabama at Birmingham, Birmingham, AL, United States; ^3^ Department of Radiation Oncology, Ohio State University, Columbus, OH, United States

**Keywords:** SBRT (stereotactic body radiation therapy), breast cancer, accelerated partial breast irradiation (APBI), radiation oncology, prone breast irradiation, cosmesis

## Abstract

**Background:**

For appropriately selected patients with early-stage breast cancer (ESBC), accelerated partial breast irradiation (APBI) yields equivalent rates of ipsilateral breast tumor recurrence with mixed results in patient-rated cosmesis compared with whole-breast radiotherapy depending on the technique utilized. When utilizing external beam radiotherapy for APBI, techniques to reduce target margins and overall treatment volume are potentially important to decrease rates of long-term adverse cosmesis. Stereotactic body radiotherapy (SBRT) is a promising technique to deliver APBI because of its increased accuracy and sparing of uninvolved breast tissue. We report the initial results of a prospective clinical trial investigating feasibility, safety, and cosmetic outcomes of a daily five-fraction SBRT regimen for APBI.

**Methods:**

Twenty-three patients with ESBC after lumpectomy who met APBI suitability were enrolled. During lumpectomy, a bioabsorbable three-dimensional fixed array tissue marker (BioZorb™, Hologic, Marlborough, MA) was placed for enhanced visualization of the cavity boundaries. Clinical target volume (CTV) was defined as the delineable cavity plus a 1-cm isotropic expansion followed by a 3-mm isotropic planning target volume (PTV) expansion. Patients received 30 Gy delivered in five planned consecutive daily fractions in either prone or supine positioning depending on individual anatomy. Two patients completed the five-fraction treatments in 9-day interval and 11-day interval due to external circumstances. A maximum PTV of 124cc was allowed to minimize incidence of fat necrosis. Plans utilized 10-MV flattening filter–free beams delivered on a Varian Edge linear accelerator. Local control, toxicity, and nurse/patient-scored cosmesis at pre-treatment baseline, 1 month post-treatment, and at subsequent 6-month intervals were recorded.

**Results:**

Twenty-three patients were accrued at the time of submission with median follow-up of 6 months. No patients experienced grade ≥3 acute toxicity. Of the 10 events reported probably related to SBRT, nine were grade 1 (n = 9/10, 90%). There was no evidence of difference, deterioration, or change in patient or nurse-scored cosmesis from baseline to 1 and 6 months post-treatment. One patient developed nodal failure shortly after APBI.

**Conclusions:**

Although longer follow-up is needed to assess long-term toxicity and local control, this study demonstrated a five-fraction SBRT regimen delivered over consecutive days is a safe, efficient, well-tolerated, and cosmetically favorable means of delivering APBI in suitable women.

**Clinical Trial Registration:**

https://www.clinicaltrials.gov/ct2/show/NCT03643861, NCT03643861.

## Introduction

For patients with breast cancer who pursue breast conservation, adjuvant radiation therapy after lumpectomy has been shown to reduce the risk of local recurrence and improve overall survival (1). With improved detection strategies and screening programs, the incidence of early-stage breast cancer (ESBC) is increasing. Treatment for patients with ESBC typically begins with the surgical decision of either mastectomy or breast conservation surgery (BCS). Although BCS consisting of lumpectomy and adjuvant whole-breast irradiation (WBI) has been shown to have similar long-term survival compared with mastectomy for patients with ESBC, approximately one of three women undergo mastectomy (2, 3). Some contributing factors to treatment decision include cosmesis, breast reconstruction, duration of radiation treatment course, and possible radiation adverse effects.

Accelerated partial breast irradiation (APBI) is becoming a more common alternative to WBI for adjuvant radiation in the BCS setting. In APBI, radiation is delivered to only part of the breast, specifically the lumpectomy cavity with a defined treatment margin (4). The foundation of APBI is due to a study by Vicini et al., who performed a pathologic analysis of residual disease on re-excision after lumpectomy and found over 90% of patients with disease fell within 1cm of the lumpectomy cavity (5). Other studies show that, compared with WBI, APBI has reduced treatment time and spares healthy breast tissue while maintaining similar local control and overall survival in select patients, which are defined by the American Society of Therapeutic Radiology and Oncology (ASTRO) guidelines (4). A variety of techniques are available for APBI, each associated with potentially increased occurrence of particular adverse effects (4). Fractionated three-dimensional (3D) external beam radiation therapy (EBRT), unlike brachytherapy, is non-invasive but has been associated with poor cosmesis (6, 7). Stereotactic body radiation therapy (SBRT) is a newer technique for APBI, which may offer a non-invasive option with improved cosmesis. SBRT uses unique planning and immobilization techniques to deliver higher doses of radiation per fraction with less total treatments (8). SBRT could provide a non-invasive option with decreased treatment time, decreased toxicity to healthy tissues, and better cosmesis than other APBI techniques. SBRT has been used for many years in other anatomical sites; however, its use in breast cancer has not been widely studied. Timmerman conducted one of the first breast SBRT studies and helped define radiation doses and target as well as associated adverse events (8). More recently, Brunt et al. published a non-inferiority study looking at outcomes and cosmesis results comparing five-fraction whole-breast radiation therapy regimens with a standard 15-fraction regimen (9). This study showed non-inferiority in recurrence and survival for two five-fraction regimens, 26 Gy in five fractions and 27 Gy in five fractions (9). Cosmesis was worse for 27 Gy in five fractions, but not 26 Gy in five fractions (9). SBRT could potentially allow maintenance of recurrence and survival compared with whole-breast RT while improving cosmesis due to decreased tissue irradiated. SBRT, in particular, could allow decreased margins compared with non-SBRT techniques to further minimize normal breast tissue dose, which could lead to improved cosmesis. In this pilot study, we investigated the safety, feasibility, and radiation target of SBRT in breast cancer using a traditional linac platform. Radiation prescription was 30 Gy in five fractions treated on consecutive days for 91% of patients. A 3D fiducial marker was placed in the lumpectomy cavity to aid in target delineation. Acute and late toxicities as well as patient and nurse cosmesis were tracked. Preliminary results are reported here.

## Materials and Methods

### Patient Eligibility

We enrolled 23 clinically and pathologically node negative patients with ESBC after lumpectomy who met APBI suitability: age ≥50, ER+, margins negative by at least 2 mm if invasive histology or 3 mm if DCIS, Tis, or T1; if DCIS, detected on screening mammogram, grades 1–2, and size<2.5 cm (4). One patient had both right and left breasts treated; there were therefore 24 separate treatment plans with 23 patients. Invasive histology meeting eligibility included ductal, medullary, papillary, mucinous (colloid), and tubular. Patients were clinically node negative by physical examination. Of the patients who had sentinel node dissection performed, all were pathologically node negative. Patients were allowed to receive adjuvant chemotherapy and endocrine therapy at the discretion of the treating physician. Demographic data, including age, race, ethnicity, weight, height, and BMI were recorded (**Table 1**). Exclusion criteria included multifocal or multicentric cancer, receiving neoadjuvant chemotherapy, pure invasive lobular histology, surgical margins < 2 mm, inability to clearly delineate lumpectomy cavity on post-lumpectomy planning scan, measured maximum PTV of >124cc, and lumpectomy cavity within 5 mm of body contour on the treatment planning system.

**Table 1:** Patient demographics, treatment characteristics, and location of breast cancer.

Characteristics	n	Mean ± 1SD	Minimum–Maximum
Age (years)	23	60.7 ± 6.93	50–76
Height (in)	22	64.6 ± 2.93	59.0–70.0
Weight (kg)	22	77.4 ± 19.5	45.8–121
BMI (kg/m^2^)	22	28.8 ± 7.49	17.9–45.8
PTV (cm^3^)	24	80.8 ± 24.3	50.3–124
GTV (cm^3^)	24	8.51 ± 3.38	3.90–16.5
CTV (cm^3^)	24	51.6 ± 17.8	24.2–87.3
Total Ipsilateral Breast Volume (cm^3^)	24	1040 ± 578	211–2,320
**Race**	**Frequency**	**Percent (%)**
African American	4	17.4	
Caucasian	17	73.9	
Declined to Answer	1	4.35	
Unknown	1	4.35	
**Ethnicity**			
Non-Hispanic/Latino	23	100	
**Quadrant of Breast Cancer**			
Lower (6:00)	2	8.33	
Lower Inner (4:00)	1	4.17	
Upper (12:00)	2	8.33	
Upper Inner	4	16.7	
Upper Inner (11:00)	2	8.33	
Upper Inner (9:00)	2	8.33	
Upper Outer	2	8.33	
Upper Outer (10:00)	1	4.17	
Upper Outer (11:00)	3	12.5	
Upper Outer (12:00)	1	4.17	
Upper Outer (1:00)	1	4.17	
Upper Outer (3:00)	2	8.33	
Upper Outer (near 3:00)	1	4.17	

### Planning and Treatment

During lumpectomy, a breast surgeon placed a bioabsorbable 3D fixed array tissue marker (BioZorb™, Hologic, Marlborough, MS) for enhanced visualization of the cavity boundaries. The array marker consists of six titanium clips connected by a bioabsorbable polylactic acid. The marker was sized by the surgeon based on the expected lumpectomy cavity volume and was sutured into the cavity (10).

Initially, all patients were treated in the prone position due to concerns for respiratory induced chest wall motion in the supine position. However, as the study progressed, many patients preferred the supine position due to discomfort with prone immobilization. Sixteen patients, 17 plans due to one patient with bilateral breast cancer, were treated in the prone position using the QFix immobilization system without respiratory motion management. Seven patients were treated in the supine position. Of the seven patients, one was treated with deep inspiration breath hold (DIBH), whereas the other six were treated using the free breathing.

**Figure 1:**
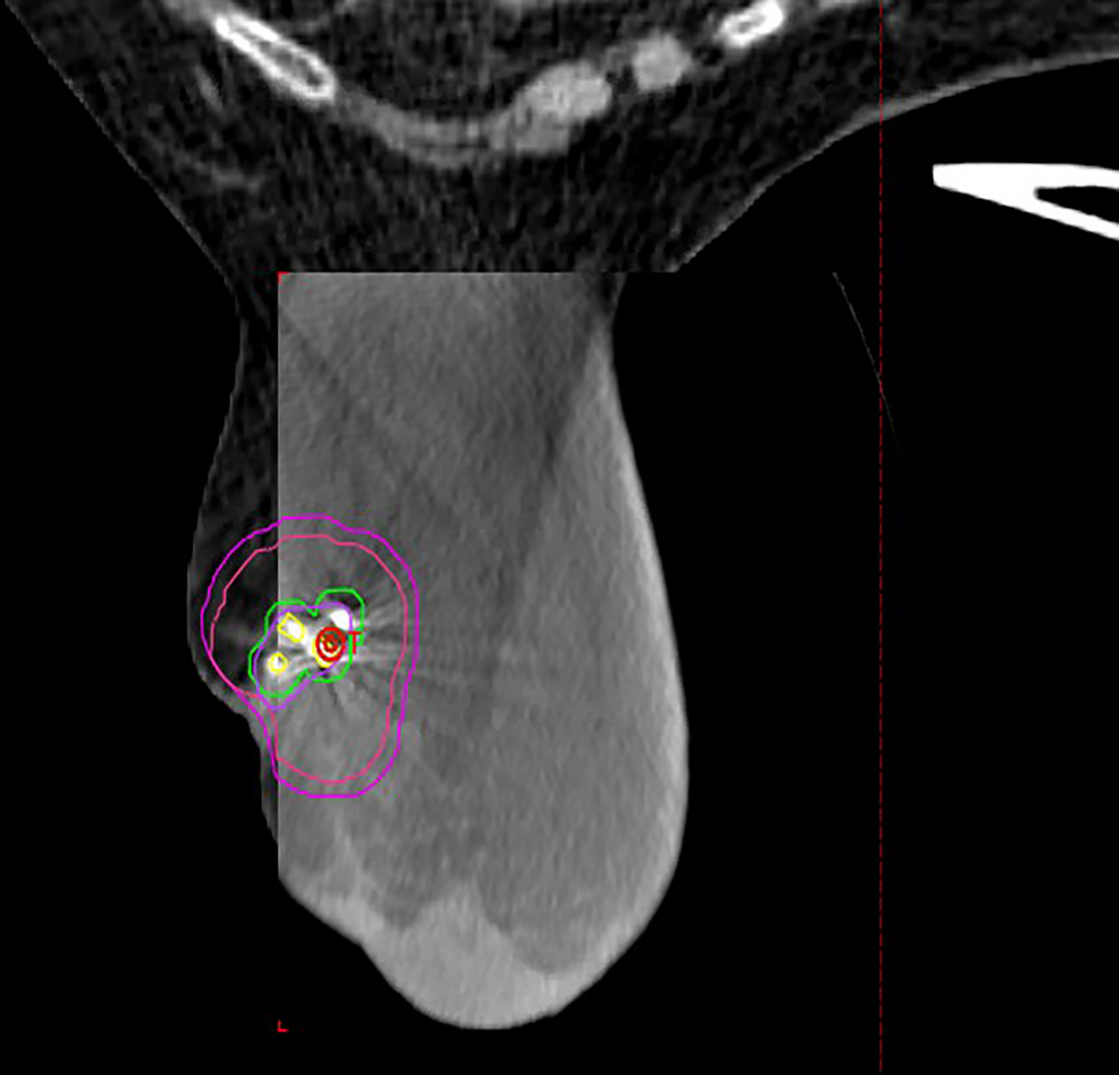
Representative patient treated in the prone position with Biozorb™ fiducials contoured in yellow with 3-mm margin for fiducial tracking. Lumpectomy cavity is contoured in purple with representative CTV (red) and PTV (Magenta) expansion.

**Figure 2:**
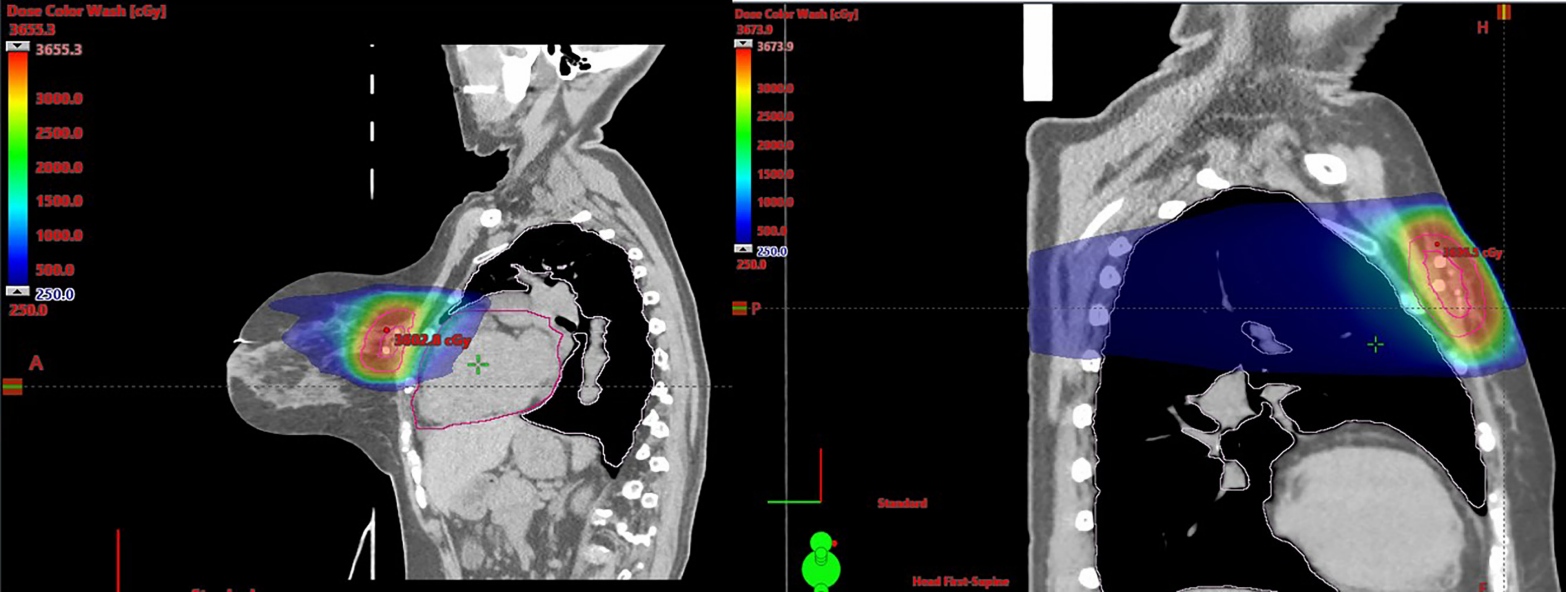
Representative images of the same patient planned in both the supine and prone position.

The lumpectomy cavity was delineated with the aid of the breast surgeon and 3D marker. The cavity was defined as gross tumor target volume (GTV). A clinical target volume (CTV) of 1 cm was expanded from the GTV and carved off natural boundaries such as rib, muscles, and lungs. CTV was cropped 5 mm off the skin. A planning target volume (PTV) of 3 mm was added to CTV concentrically (**Figure 1**). Normal structures included the heart, left lung, right lung, ipsilateral breast, contralateral breast, skin, and rib within 5 cm of PTV boundary. All patients met protocol-specific constraints as shown in **Supplementary Table 1**. The prescription dose was 30 Gy delivered once daily to PTV for a total of five fractions at 6 Gy per fraction. Fourteen patients completed treatments in 5 days, seven patients completed the treatments in 7 days due to interference of weekends, one patient completed treatments in 9 days due to technical issues, and one patient completed treatments in 11 days due to hazardous weather. Thus, 91% of patients were treated in consecutive days. All SBRT treatments were delivered on the Varian Edge linear accelerator. Prone plans were delivered with 7–13 co-planar IMRT fields (median MUs/fx = 2,189) with 10-MV flattening filter–free beams. Supine plans were delivered using static fields (n = 3 plans, eight to nine beams, and MU/fx) and VMAT (n = 4 plans, two partial arcs per plan). **Figure 2** shows representative plans in a single patient planned in both the supine and prone position. Daily imaging included orthogonal kV images aligning to 3D tissue marker contoured on the planning CT scan followed by a cone beam CT to ensure no seed migration or a significant change in seroma volume had occurred. Triggered imaging was taken prior to each couch shift to ensure fiducials were within the 3 mm of region of interest prior to treatment delivery. During treatment, real-time kV images were triggered by gantry motion (10°) for VMAT or by time (3 s) for static IMRT. One patient was treated with DIBH, which utilized Varian RPM, because her heart was very close to chest wall. All other patients were treated using free breathing. Beams were put “on-hold” when fiducial marker excursions exceeded 3 mm.

### Follow-Up

The objective of the study was to accrue 20 patients and evaluate cosmesis, acute and chronic adverse events, and disease recurrence. Adverse events were based on Clinical Terminology Criteria for Adverse Events (CTCAE 4.0) and causality of the event to SBRT. Cosmesis was scored by both nurse and patient (i.e., 1 = excellent, 2 = good, 3 = fair, and 4 = poor). Photographs of the treated breast were taken pre-treatment, 1-month post-treatment, and at each subsequent 6-month interval up to 3 years after treatment completion. Acute toxicity was defined as toxicity ≤90 days, and late toxicity was defined as toxicity >90 days. Causality of the event to SBRT was designated as seen in **Supplementary Table 2**. This included unrelated, unlikely, possible, probable, and definite, whereas some adverse events were undesignated. Serious adverse events were defined as grade 3 toxicity deemed definitely related to treatment in the following categories: skin, rib (fracture), pulmonary (radiation pneumonitis), neurologic (intercostal), or any grade 4 to 5 toxicity deemed definitely attributed to radiation. Cosmesis scores were graded as seen in **Supplementary Table 3**. Nurses recorded treatment effects described in **Supplementary Table 4**. In addition, mammogram was ordered at approximately 6, 18, and 36 months post-surgery.

### Statistics

To access central tendency and dispersion for continuous variables such as age of subject, sample means and standard deviations were calculated. To summarize categorical variables such as race, the proportion of subjects having the specific characteristic was reported. To test whether patient and nurse reports of cosmesis differed significantly, the Wilcoxon-signed rank test was used given the ordinal nature of the outcomes. Similarly, to determine whether reported cosmesis changed over time, Friedman’s test was used to account for the ordinal nature of the outcomes. All tests were conducted in SAS 9.4 using a two-tailed type I error rate of 0.05.

## Results

Between August 20, 2019, and April 27, 2021, 23 patients were accrued to the study at the time of submission with median follow-up of 6 months. One of the 23 patients had left and right breasts treated, yielding 24 treatment plans total. Seventeen treatments were performed in the prone position using free breathing. Seven treatments were in the supine position. Of the treatments, one was planned and treated with breath hold because her heart was very close to chest wall, whereas 6 treatments utilized free breathing. There were 12 left breast treatments and 12 right breast treatments. Demographic data including age, race, ethnicity, weight, height, and BMI were reported (**Table 1**). Notable medical history of the patients included diabetes mellitus in three patients, Raynaud’s syndrome in one patient, Hashimoto thyroiditis in one patient, systemic lupus erythematosus in one patient, and Sjogren’s syndrome in one patient. GTV, CTV, PTV, and total ipsilateral breast volume are detailed in **Table 1**, with mean PTV of 80.76 cm^3^. Upper outer quadrant of the breast was the most common location of breast cancer (**Table 1**).

### Cosmesis

**Figure 3:**
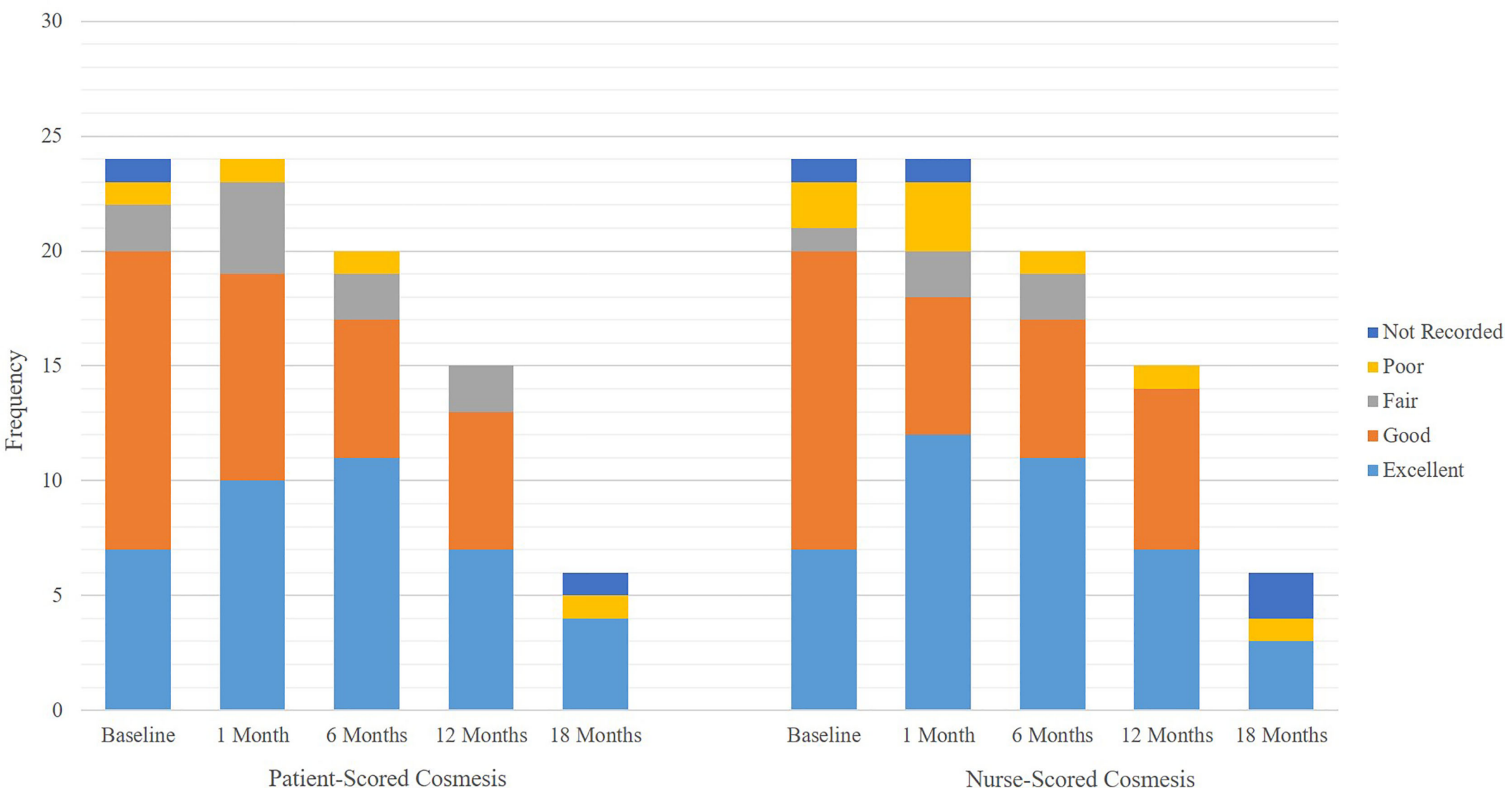
Frequency of patient scored cosmesis versus nurse scored cosmesis at baseline, 1, 6, 12, and 18 months.

**Figure 4:**
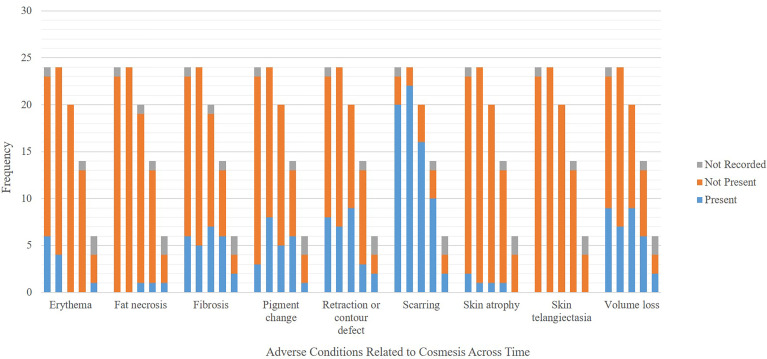
Frequency of adverse conditions related to cosmesis. In a category, each bar on the x-axis represents scores at (from left to right) baseline, follow-up at 1, 6, 12, and 18 months.

At time of submission, 19 patients completed the 6-month follow-up and 13 patients completed the 12-month follow-up. A total of 87% of patient-scored cosmeses were excellent or good at baseline, 79% at 1 month, and 85% at 6 months (p = 0.622 for change from baseline to 6 months). There was no evidence of deterioration or change in patient-scored cosmesis from baseline to 6 months post-treatment. A total of 87% of nurse-scored cosmeses were excellent or good at baseline, 78% at 1 month, and 85% at 6 months (p = 0.0783 for change from baseline to 6 months). There was no evidence of deterioration or change in nurse-scored cosmesis from baseline to 6 months post-treatment. There was no difference in patient-scored cosmesis and nurse-scored cosmesis at baseline, 1 month, and 6 months; all respective p-values are equal to 1. Detailed patient-scored cosmeses and nurse-scored cosmeses at baseline through 18 months post-SBRT can be found in **Figure 3**. Fat necrosis was not present in 100% of patients at 1 month post-SBRT, whereas one patient had fat necrosis present at 6, 12, and 18 months (**Figure 4**). Detailed nurse-evaluated adverse effects can be found in **Figure 4**, with scarring being the most frequently identified at baseline through 18 months post-SBRT; the frequency of patients with scarring decreased from 1-month post-SBRT.

### Adverse Events

Adverse events reported probable to SBRT were mostly grade 1 (n = 9/10, 90%). The only grade 2 adverse event probable to SBRT was generalized arthralgia, and the only possible adverse event due to SBRT was fatigue (n = 2). All adverse events can be found in **Supplementary Tables 5, 6**. Grade 1 adverse events with probable relationship to SBRT included fatigue, generalized arthralgia, and skin hyperpigmentation with frequency of 2, 2, and 3, respectively. There were no grade ≥3 toxicities. Thirteen patients reached the 12 month follow-up mark, and there have been no serious adverse events after SBRT for any patients. Although follow-up is too short to make definitive conclusions on recurrence, there have been no ipsilateral breast cancer recurrences. However, one patient had an ipsilateral axillary nodal failure of 3 cm in size and was treated with chemotherapy, followed by salvage comprehensive nodal radiotherapy.

## Discussion

This pilot study shows the feasibility and safety of breast SBRT with a median follow-up of 6 months. APBI continues to increase in popularity for both patients and physicians due to reduced treatment duration while potentially maintaining a favorable acute toxicity profile and cancer outcomes. Many different treatment modalities exist such as brachytherapy, SBRT with robotic radiosurgery system, and SBRT with a traditional linac platform as investigated in this study. Despite many advances in technology, cosmesis concerns and toxicity continue to be an obstacle for wide acceptance of APBI. As such, this was a chief focus in our pilot study, using prior studies as a guide for toxicity expectations and objectives. Gabani et al. looked into APBI using multi-catheter interstitial brachytherapy treating 34 Gy in 10 twice-daily fractions to 2 cm of breast tissue surrounding the surgical bed. Grade 1 or 2 skin toxicity was reported in 44/175 patients, and grade 3 toxicity in 1/175 patients (11). Cosmesis outcomes were excellent in 66%, good in 29%, fair in 5%, and poor in 0% (11). External beam APBI utilizing 3D techniques eliminates the invasiveness of brachytherapy but has not shown improved skin toxicity and cosmesis. This may be related to larger treatment volumes due to target margins and subsequent challenges meeting dose constraints. In a study by Hepel et al., 3D CRT conformal radiotherapy (CRT) was used for APBI in 60 patients with cosmesis outcome of either good or excellent in 81.7%, fair in 11.7%, and poor in 6.7% (12). The risk of poor cosmesis and development of subcutaneous fibrosis was correlated to the ratio of volume of breast tissue receiving 5% and 20% of the prescription dose to the whole-breast volume (12). When external beam radiation was given at 38.5 Gy in 10 fractions delivered twice per day over 5–8 days in a study by Whelan et al., fair or poor cosmesis was more common in patients treated with APBI than in those treated by whole-breast irradiation at 3, 5, and 7 years, with authors attributing poor cosmesis to the twice per day irradiation (13). Livi et al. used 30 Gy in five daily fractions delivered with IMRT to the tumor bed compared with 50 Gy in 25 fractions in the WBI arm, with an additional 10-Gy boost on the tumor bed in five fractions (14). The IMRT group had significantly better acute toxicity (p = 0.0001), late toxicity (p = 0.004), and cosmetic outcome (p = 0.045) (14). Rahimi et al. used a robotic radiosurgery platform in a dose escalation study, starting at 30 Gy in five fractions and escalating by 250 cGy up to 40 Gy; this study recommended the cutoff PTV volume of 124 cm^3^ that was used in our study, due to increased likelihood of fat necrosis beyond that volumetric threshold (8). The cosmesis score was excellent or good in 86.5% at baseline, 97.1% at 6 months, 95.1% at 12 months, and 95.3% at 24 months, with patients reporting improved cosmesis within the first year (8). This showed promising cosmesis with reduced treatment duration but potentially limited practicality due to availability of robotic radiosurgery systems. SBRT studies in ESBC have also included preoperative radiation in a single fraction to address the poor cosmesis (15, 16). Both studies showed feasibility of SBRT. In another preoperative SBRT study, radiation was given concurrently with neoadjuvant chemotherapy using five different doses to evaluate maximum dose tolerance, showing feasibility of SBRT at a dose as high as 31.5 Gy in three fractions (17).

These studies helped shape our protocol for determining radiation prescription and target volume. Patient selection is a necessary initial step in breast SBRT. In this study, ASTRO’s 2017 updated consensus statement for APBI was used and followed for patient selection (4). Patients younger than 50 were deemed to be at a higher risk for recurrence using partial breast irradiation as opposed to whole-breast radiation, thus were excluded from this study. Unique aspects of this study include the utilization of a 3D fiducial marker was placed in the lumpectomy cavity at time of resection to aid with localization of target, individualized patient positioning decision supine vs. prone to minimize dose to organs at risk, and further accelerated treatment regimen using a dose prescription of 30 Gy in five fractions instead of every other day to consecutive days to improve patient convenience. We found the fiducial marker facilitated lumpectomy cavity delineability, which increased localization confidence and minimized treatment margins. Utilizing a fiducial was also found to be useful for intrafraction motion monitoring, which further increased confidence in minimizing margins. This could help with practical aspects of the surgery with fiducial placement. Given continued tracking intrafractionally, margins are still able to be minimized. Provided cosmesis and adverse effects have been related to target volume in previously discussed studies, increasing localization confidence with intrafraction verification should lend to favorable outcomes.

Whereas five-fraction whole-breast regimens such as described in the Fast Forward study are clinically acceptable when compared with hypofractionated whole-breast radiotherapy, there are clinically significant increases in breast edema and breast induration (outside the tumor bed) in the 26Gy in five-fraction arm compared with the 40 Gy in 15-fraction arm (9). As such, SBRT has the potential to further reduce toxicity and improved cosmesis compared with treating larger volumes, although this would need to be evaluated in a randomized study. In addition, APBI has been modeled to reduce the risk of radiation related secondary malignancy compared with whole-breast RT due to reduction in integral dose to the heart and lung (18). SBRT in particular is an attractive technique for APBI as it raises the potential to further reduce the number of fractions while preserving cosmesis (19).

Preliminary, 1-month results in our study showed safety and feasibility of five-fraction SBRT delivered on a traditional linear accelerator platform, with excellent or good patient and nurse reported acute cosmesis. None of the patients developed grade 3 or greater acute toxicity related to radiation treatment within the 1-month follow-up period. Our patient population helps to further define and validate the selection criteria for APBI with SBRT. We will continue to monitor for cosmesis scores and adverse events up to 3 years. Limitations of this study are the pilot nature of limited 23-patient participation and the short follow-up period for cosmesis and adverse events. In addition, variable cosmetic scoring criteria between studies make comparison with previously published historical controls difficult. Longer follow-up and increased participants are needed to follow late toxicities and cosmesis scores.

In conclusion, breast APBI with SBRT using 30 Gy in five fractions delivered on consecutive days with a traditional linac platform was found to result in excellent patient and nurse reported cosmesis with minimal adverse events at 1-month follow-up. Utilizing a 3D fiducial marker facilitated lumpectomy cavity delineability and helped decrease target volume by minimizing margins. The addition of prone positioning facilitated lower dose to critical structures in some patients, potentially reducing adverse events. Some patients were found to benefit more with supine positioning, which will be reported separately. We will continue to follow-up the patients in this study for long-term toxicity, local control, and survival outcomes.

## Data Availability Statement

The raw data supporting the conclusions of this article will be made available by the authors, without undue reservation.

## Ethics Statement

The studies involving human participants were reviewed and approved by University of Alabama Birmingham IRB. The patients/participants provided their written informed consent to participate in this study.

## Author Contributions

YL and CV analyzed data and prepared the manuscript. DH gathered and analyzed data and prepared the manuscript. HK enrolled patients and contributed to manuscript preparation. AD and KM participated in treatment planning and manuscript preparation. MD, RL, MB, CP, and KK enrolled patients and contributed to manuscript preparation. ET conceptualized the study, analyzed data, and contributed to manuscript preparation. DB conceptualized the study, enrolled patients, analyzed data, oversaw and contributed to manuscript preparation. All authors contributed to the article and approved the submitted version.

## Funding

This study was funded by Varian Medical Systems Inc.

## Conflict of Interest

DB, ET, and MB disclose Varian Medical Systems sponsored honoraria and research funding. DB discloses Novocure Inc.–sponsored consulting and research support. MB discloses NIH and NIND funding.

The authors declare that this study received funding from Varian Medical Systems Inc. The funder had the following involvement in the study: study design.

The remaining authors declare that the research was conducted in the absence of any commercial or financial relationships that could be construed as a potential conflict of interest.

## Publisher’s Note

All claims expressed in this article are solely those of the authors and do not necessarily represent those of their affiliated organizations, or those of the publisher, the editors and the reviewers. Any product that may be evaluated in this article, or claim that may be made by its manufacturer, is not guaranteed or endorsed by the publisher.
