# Supplementary material for: Feasibility and Short-Term Toxicity of a Consecutively Delivered Five Fraction Stereotactic Body Radiation Therapy Regimen in Early-Stage Breast Cancer Patients Receiving Partial Breast Irradiation
Source: Front Oncol. 2022 Jul 8;12:901312. doi: 10.3389/fonc.2022.901312 (PMC9307906; doi:10.3389/fonc.2022.901312)
Supplement: Supplementary file 1 [file DataSheet_1.docx]

**Supplementary Table 1.** Dose constraints

| Organ | Volume | Dose | Maximum  Point dose (Gy) | V100 (cm3) | Conformality  Index |
| --- | --- | --- | --- | --- | --- |
| - Ipsilateral Lung | - <10% | - 9Gy |  |  |  |
| - Contralateral Lung | - <10% | - 1.5Gy |  |  |  |
| - Heart (right sided lesion) | - <5% | - 1.5Gy |  |  |  |
| - Heart (left sided lesion | - <40% | - 1.5Gy |  |  |  |
| - Skin | - <10cc | - 36.5Gy | - 39.5 |  |  |
| - Ipsilateral Breast | - <40% - <20% | - 15Gy - 30Gy |  |  |  |
| - Contralateral Breast | - < .03cc - <20% | - 15Gy - 5Gy |  |  |  |
| - Rib |  |  | - <43 | - <1 |  |
| - PTV |  |  |  |  | - <1.3 |

**Supplementary Table 2.** Causality relationship of the radiosurgery to a serious adverse event

| Causality | Definition |
| --- | --- |
| Unrelated | There is no evidence of causal relationship |
| Unlikely | There is *little* evidence to suggest there is a causal relationship (*e.g.,* the event did not occur within a reasonable time after administration of the radiosurgery). There is *another reasonable explanation* for the event (e.g., the pat*ient’s clinical condition, oth*er concomitant treatments). |
| Possible | There is *some* evidence to suggest a causal relationship (e.g., the event occurred within a reasonable time after administration of the radiosurgery). However, the influence of *other factors may have contributed* to the event (e.g., the patient’s clinical condition, other concomitant events). |
| Probable | There *is evidence* to suggest a causal relationship, and the influence of other factors is *unlikely*. |
| Definite | There is clear evidence to suggest a causal relationship, and other possible contributing factors can be ruled out. |

**Supplementary Table 3.** Definitions of the Cosmesis Scores

| Score | Definition |
| --- | --- |
| 1 | **EXCELLENT:** When compared with the untreated breast or the original appearance of the breast, there is minimal or no difference in the size or shape of the treated breast. The way the breast feels (its texture) is the same or slightly different. There may be thickening, scar tissue, or fluid accumulation within the breast, but not enough to change the appearance. |
| 2 | **GOOD**: There is slight difference in the size or shape of the treated breast as compared with the opposite breast or the original appearance of the treated breast. There may be some mild reddening or darkening of the breast. The thickening or scar tissue within the breast causes only a mild change in the shape or size. |
| 3 | **FAIR**: Obvious differences in the size and shape of the treated breast. This change involves one-quarter or less of the breast. There can be moderate thickening or scar tissue of the skin and the breast, and there may be obvious color changes. |
| 4 | **POOR**: Marked change in the appearance of the treated breast involving more than one quarter of the breast tissue. The skin changes may obvious and detract from the appearance of the breast. Severe scarring and thickening of the breast, which clearly alters the appearance of the breast, may be found. |

**Supplementary Table 4.** Specific treatment effects in relation to cosmesis

| **Treatment effect** | **Treatment effect** | **Yes, present but does not affect cosmesis** | **Yes, present and affects cosmesis** |
| --- | --- | --- | --- |
| Skin telangiectasia | 0 | 1 | 2 |
| Skin atrophy | 0 | 1 | 2 |
| Scarring | 0 | 1 | 2 |
| Pigment change | 0 | 1 | 2 |
| Erythema | 0 | 1 | 2 |
| Fat necrosis | 0 | 1 | 2 |
| Fibrosis | 0 | 1 | 2 |
| Retraction or contour defect | 0 | 1 | 2 |
| Volume loss | 0 | 1 | 2 |
| Other significant treatment effects  Specify: | | | |

| **Supplementary Table 5.** Adverse events with undesignated, possible, probable, and unlikely relationship to SBRT | | |
| --- | --- | --- |
| **Adverse Events: Undesignated** | **Frequency** | **Percent (%)** |
| Abdominal skin biopsy | 1 | 3 |
| Ankle edema, bilateral | 1 | 3 |
| Blood & lymphatic system disorder other (lymphedema syndrome) | 1 | 3 |
| Breast hyperpigmentation | 1 | 3 |
| Breast pain | 2 | 6 |
| Breast pruritis, left | 1 | 3 |
| Breast tenderness, right | 1 | 3 |
| Breast tenderness, left | 1 | 3 |
| Breast tenderness, right | 1 | 3 |
| Chills | 1 | 3 |
| Constipation | 1 | 3 |
| Dermatitis (radiation) | 1 | 3 |
| Dermatitis, underside breast bilateral | 1 | 3 |
| Dry skin | 1 | 3 |
| Dysthermia | 1 | 3 |
| Fatigue | 5 | 16 |
| Generalized muscle weakness | 1 | 3 |
| Hepatic cysts, left lobe | 1 | 3 |
| Hot flashes | 2 | 6 |
| Hyperpigmentation, left nipple | 1 | 3 |
| Hyperpigmentation, rt breast | 1 | 3 |
| Hypersomnia | 1 | 3 |
| Liver hemangioma | 1 | 3 |
| Nausea | 1 | 3 |
| Non-cardiac chest pain | 1 | 3 |
| Vaginal hemorrhage | 1 | 3 |
| **Adverse Event: Possible** |  |  |
| Fatigue | 2 | 100 |
| **Adverse Events: Probable** |  |  |
| Dermatitis (radiation) | 1 | 10 |
| Fatigue | 2 | 20 |
| Generalized arthralgia | 1 | 10 |
| Generalized arthralgia | 1 | 10 |
| Hyperpigmentation, rt breast | 1 | 10 |
| Nausea | 1 | 10 |
| Skin hyperpigmentation | 1 | 10 |
| Skin hyperpigmentation, left breast | 1 | 10 |
| Skin hypopigmentation | 1 | 10 |
| **Adverse Events: Unlikely** |  |  |
| Breast pain, rt | 1 | 25 |
| Breast rash, bilateral | 1 | 25 |
| Hot flashes | 1 | 25 |
| Non-cardiac chest pain | 1 | 25 |

| **Supplementary Table 6.** Adverse events with unrelated relationship to SBRT | | | |
| --- | --- | --- | --- |
| **Adverse Events: Unrelated** | | **Frequency** | **Percent (%)** |
| Abscess, rt index finger | | 1 | 1 |
| Abdominal tenderness | | 1 | 1 |
| Alopecia | | 1 | 1 |
| Anxiety | | 2 | 2 |
| Arthralgia | | 4 | 4 |
| Arthralgia, bilateral hands | | 1 | 1 |
| Arthralgia, hands & back | | 1 | 1 |
| Axillary paresthesia | | 1 | 1 |
| Back pain | | 1 | 1 |
| Bilateral fallopian tubes & ovaries removed | | 1 | 1 |
| Bilateral hearing loss | | 1 | 1 |
| Breast biopsy, left | | 1 | 1 |
| Breast hamartoma, rt | | 1 | 1 |
| Breast seroma, left | | 1 | 1 |
| Breast volume decreased, rt | | 1 | 1 |
| Bullous dermatitis | | 1 | 1 |
| Carpal tunnel syndrome, rt upper limb | | 1 | 1 |
| Clavicle surgery, rt | | 1 | 1 |
| Constipation | | 1 | 1 |
| Cough | | 3 | 3 |
| Decreased smell | | 1 | 1 |
| Diarrhea | | 1 | 1 |
| Dysgeusia | | 1 | 1 |
| Facial telangiectasia | | 1 | 1 |
| Fever | | 1 | 1 |
| Finger paresthesia | | 1 | 1 |
| Flu-like symptoms | | 1 | 1 |
| Flu-like symptoms (covid-19) | | 1 | 1 |
| Gastroesophageal reflex | | 1 | 1 |
| Hearing impairment, bilateral | | 1 | 1 |
| Heel spurs | | 1 | 1 |
| Hematoma, rt elbow | | 1 | 1 |
| Hemorrhoids | | 1 | 1 |
| Hot flashes | 5 | | 6 |
| Hypertension | 2 | | 2 |
| Insomnia | 2 | | 2 |
| Inverted nipple, left | 1 | | 1 |
| Itchy eye, bilateral | 1 | | 1 |
| Left arm pruritis | 1 | | 1 |
| Lethargy | 1 | | 1 |
| Mouth ulcers | 1 | | 1 |
| Myalgia | 1 | | 1 |
| Nail infection, right foot | 1 | | 1 |
| Nausea | 4 | | 4 |
| Neoplasm, benign (other polyps) | 1 | | 1 |
| Neutrophil count decreased | 3 | | 3 |
| Nipple pruritis, left | 1 | | 1 |
| Osteopenia | 3 | | 3 |
| Overactive bladder | 1 | | 1 |
| Paresthesia arms & hands | 1 | | 1 |
| Rosacea paraspinal notalgia | 1 | | 1 |
| Seroma | 1 | | 1 |
| Seroma - right breast | 1 | | 1 |
| Seroma aspiration, rt breast | 1 | | 1 |
| Seroma axillary | 1 | | 1 |
| Seroma-right breast | 1 | | 1 |
| Side pain, left | 1 | | 1 |
| Sore throat | 1 | | 1 |
| Subconjunctival hemorrhage right eye | 1 | | 1 |
| Tinnitus, bilateral | 2 | | 2 |
| Trigger finger, right index | 1 | | 1 |
| Upper respiratory infection | 1 | | 1 |
| Vaginal atrophy | 1 | | 1 |
| Vomiting | 1 | | 1 |
| Weight gain | 2 | | 2 |
| White blood cell decreased | 2 | | 2 |
| Wrist fracture, rt | 1 | | 1 |
